# Evaluation of autoimmune liver disease antibodies in hepatitis patients

**DOI:** 10.1371/journal.pone.0307285

**Published:** 2024-08-20

**Authors:** Namsoo Kim, Sinyoung Kim, Jong Rak Choi, Younhee Park

**Affiliations:** 1 Department of Laboratory Medicine, Severance Hospital, Yonsei University College of Medicine, Seoul, South Korea; 2 Dxome co., Ltd., Seongnam, South Korea; University Hospital of Bologna Sant’Orsola-Malpighi Polyclinic Department of Digestive System: Azienda Ospedaliero-Universitaria di Bologna Policlinico Sant’Orsola-Malpighi Dipartimento dell’apparato digerente, ITALY

## Abstract

**Background/aims:**

Autoimmune hepatitis (AIH) is characterized by the presence of auto-antibodies and high blood immunoglobulin G (IgG) levels. In this study, the line immunoassay (LIA) was designed to assess various autoantibodies.

**Methods:**

In total, 1371 patients who underwent autoimmune liver disease antibody testing between July 2019 and November 2022 were enrolled. Autoantibodies including antinuclear antibody (ANA) and anti-mitochondrial antibody (AMA) were tested, and clinical data were collected. Statistical analyses were performed by categorizing the data based on diagnosis and IgG quantification separately. A scoring system was applied to identify individuals with AIH. Patients were also classified into the AIH and non-AIH groups.

**Results:**

The positivity rate for ANA was 80.2% in the AIH group. The IgG-high group had a high likelihood of the presence of detectable autoantibodies, with anti-Ro-52 being the most frequently detected antibody using LIA. The "Consider AIH" and "AMA" groups had 3–4 times more patients in the IgG-high group than in the "Not Considered" group.

**Conclusions:**

Among autoantibodies, the prevalence of ANA was the highest. As per LIA results, anti-Ro-52 was the most prevalent. AIH cannot be diagnosed based on IgG levels alone and must be distinguished via autoantibody testing. Therefore, extensive testing, including autoantibodies, IgG, ANA, and liver enzyme levels, will help accurately diagnose AIH.

## Introduction

Autoimmune hepatitis (AIH) is a severe, chronic liver disease characterized by the presence of autoantibodies and elevated serum immunoglobulin G (IgG) levels [1]. AIH is primarily classified into two types based on the type of autoantibodies present. Type 1 AIH is characterized by the presence of antinuclear antibodies (ANA) or smooth muscle antibodies (SMA)/anti-actin antibodies, and type 2 is characterized by antibodies against liver kidney microsome type 1 (anti-LKM1) or anti-liver cytosol type 1 (anti-LC-1) [2]. Type 1 AIH is common in adults, whereas type 2 AIH is common in children [3]. There have been AIH cases where patients were negative for ANA, SMA, and LKM1 autoantibodies but presented with other clinical characteristic features of AIH, which led to a third classification type called seronegative AIH [4]. Other autoantibodies, such as perinuclear antineutrophil cytoplasmic antibodies (p-ANCA) and antimitochondrial antibodies (AMAs), might be considered for the diagnosis of seronegative AIH [5].

Many autoantibodies are associated with AIH and primary biliary cholangitis (PBC). One of the most sensitive autoantibodies for type 1 AIH is p-ANCA [6]. AMAs can be found in patients with type 1 AIH, with a reported incidence of approximately 10% [7]. Anti-mitochondrial M2 antibody (AMA-M2) is specific to PBC; however, it can also be found in patients with AIH-PBC syndrome [8]. Antibodies against soluble liver antigen/liver-pancreas (SLA/LP) are associated with severe AIH and poor outcomes, especially in adult patients [9]. In 1988, autoantibodies for anti-LC-1 were found in 21 patients with type 2 autoimmune chronic active hepatitis [10]. Although anti-LKM-1 is frequently observed (approximately 90%), anti-LC-1 is less frequently observed (25–40%) in patients with type 2 AIH [11]. Ro-52 is an interferon-inducible protein, and antibodies against Ro-52 have been reported in some autoimmune diseases [12]. Antibodies for anti-glycoprotein-210 (gp210) are tested in patients with suspected PBC, especially those who are negative for AMA [13]. Promyelocytic leukemia protein (PML) is an autoantigen specific to PBC [14]. Anti-Sp100 antibodies are also associated with PBC [15].

Several diagnostic criteria and screening systems have been established to diagnose AIH. In the European Association for the Study of the Liver (EASL), ANA, anti-SMA, anti-LKM1, anti-LC-1, anti-SLA/LP, p-ANCA, anti-LKM3, anti-Ro-52, anti-gp210, and anti-Sp100 are included in a case-based algorithm for patients with suspected AIH or drug-induced liver injury [16]. In addition, EASL includes ANA, anti-SMA/F-actin, anti-LKM, and anti-SLA in the scoring system for AIH. Other categories included in the AIH scoring system are IgG, liver histology, and absence of viral hepatitis. Up to two scores are given for each category. A probable diagnosis of AIH is made when the total score is six, and a definite diagnosis is made when the total score is more than six [17]. According to the guidelines of the American Association for the Study of Liver Diseases (AASLD), ANA, anti-SMA, anti-LKM1, anti-SLA, p-ANCA, anti-actin, anti-LKM-3, anti-LC-1, and AMA are labeled as autoantibodies related to AIH [18].

The line immunoassay (LIA) kit has been introduced for the diagnosis of autoimmune diseases, which can be used to analyze different autoantibodies associated with these diseases and has begun to be used in clinical practice [19]. As there are various autoantibodies that need to be tested for diagnosing AIH and PBC, LIA is used to perform all tests at once. Another advantage of LIA is that it is rapid and, thus, has been widely used, especially to screen AIH.

There have been a few Korean studies on AIH, and some have focused on certain autoantibodies found in patients with AIH. The objective of this study was to determine the prevalence and positivity pattern of these antibodies and IgG using LIA and to determine the prevalence of anti-SMA, AMA, p-ANCA by IFA, and ANA. We attempted to determine the positivity patterns in the patient group in which AIH was suspected and the autoimmune liver disease antibody LIA test was requested.

## Materials and methods

### Patient data collection

Clinical data (including age, sex, diagnosis, IgG quantification, and autoantibody results) were collected from the clinical records of 1371 patients who underwent autoimmune liver disease antibody testing at Severance Hospital between July 2019 and November 2022. Patients who underwent autoimmune liver disease antibody testing were included. Patients without aspartate aminotransferase (AST)/alanine transaminase (ALT) elevation (>80 for each) and those with no history of hepatitis were excluded. Patients were diagnosed using the simplified criteria for the diagnosis of AIH [20], followed by confirmation using pathological analysis through liver biopsy. However, in unavoidable circumstances, the diagnosis was made on the basis of laboratory results alone. The data were accessed for research purposes on January 1, 2023. The study was conducted according to the Declaration of Helsinki guidelines and was approved by the Institutional Review Board of Severance Hospital, Seoul, South Korea (4-2022-1410). The need for informed consent was waived given the retrospective nature of the study (review of medical records) on the condition that the study involved no more than minimal risk to the patients and that their privacy was thoroughly protected.

### Autoantibody measurements

Autoantibody detection using Hep-2 cells was performed using a NOVA Lite^®^ ANA Kit (INOVA Diagnostics, Inc., San Diego, CA, USA) for the detection of ANA. Samples were diluted to an initial titer of 1:80 using a specimen diluent, according to the manufacturer’s instructions. The p-ANCA detection tests were performed using a NOVA Lite^®^ ANCA (Ethanol) Kit (INOVA Diagnostics, Inc.). For this step, patient samples were diluted 1:20 using the specimen diluent, according to the manufacturer’s instructions. The slides were evaluated using the NOVA View software, and the results were manually reviewed by two experts. Anti-SMA testing was performed using a NOVA Lite^®^ ANA KSL Mouse Kidney/Stomach/Liver kit (INOVA Diagnostics, Inc.). The screening dilution for SMA was 1:20. All test slides for SMA tests were manually analyzed by a laboratory medicine specialist using a fluorescence microscope.

To detect anti-SLA/LP, anti-LC-1, anti-LKM-1, AMA-M2, anti-Sp100, anti-PML, anti-gp210, and anti-Ro-52 antibodies, LIA was performed using the Euroline autoimmune liver diseases assay (Euroimmune, Lübeck, Germany), according to the manufacturer’s instructions. Serum was diluted 1:101 and incubated with a strip for 30 min at 20–25°C, followed by reaction with a working-strength enzyme in an incubation channel for 30 min at room temperature and a final 10-min substrate incubation at room temperature. After drying, the strips signal intensities were evaluated with EUROLineScan (Euroimmune).

### Statistical analysis

We classified the collected data by diagnosis. For patients who underwent IgG quantification, autoantibodies were analyzed for each of the three groups: IgG-low, less than the upper limit; IgG medium, <1.1 times the upper limit; and IgG-high, >1.1 times of the upper limit. p-value was obtained using Pearson’s chi-square test. We set three criteria to distinguish the AIH candidate group from patients with no liver biopsy: absence of viral hepatitis (absence of hepatitis A, B, and C viruses), classification in the IgG-high group, and positivity for at least one autoantibody. Each fulfilled condition scored one point, and the patients were classified by score. We also classified patients into the "Consider AIH" and "Not Considered" groups according to the EASL criteria [16] and further classified AMA-M2–positive patients into an "AMA" group to compare the AST, ALT, gamma-glutamyl transferase (gamma-GT), and IgG quantification values.

Microsoft Office Excel version 2211 (Microsoft Co., Redmond, WA, USA) and IBM SPSS Statistics 26 (IBM corp., Armonk, MY, USA) were used for all statistical analyses.

## Results

### Demographic features

In total, 1371 patients with hepatitis and suspected to have AIH were tested using the LIA, and collected data were classified by clinical diagnosis (Table 1). In addition, sex; age; the results of ANA testing by Hep-2 cell detection; anti-SMA and p-ANCA antibody levels; and IgG quantification were evaluated. Although most patients were tested for cytoplasmic ANCA (c-ANCA), c-ANCA was not included in the analysis as it is not part of any guidelines. Of the patients tested using the LIA, 117 had AIH, 192 had nonalcoholic steatohepatitis, 320 had viral hepatitis, and 742 were classified as having another hepatitis. The tested patient group had more females than males; the female-to-male ratio was as high as 3:1 in the AIH group. The mean age was ~50 years (49.7–56.0 years) and was similar for each group. For ANA identified using Hep-2 cells, the positivity rate was 80.2% in the AIH group, which was more than twice that in the other groups. For anti-SMA identified using the indirect immunofluoresence assay (IIF), the positivity rate in the AIH group (26.1%) was higher than that in the other groups (9.2–13.9%). A positivity rate of 33.8% was observed for p-ANCA in the AIH group; this rate was 2–3 times higher than that in the other groups. The three antibodies anti-SLA/LP, anti-LC-1, and anti-LKM-1 were rarely found in the AIH group. AMA-M2 was detected in the AIH group at a frequency of 21.4%, and among the 25 patients in whom it was detected, seven were diagnosed with concomitant PBC and AIH. Anti-Ro-52 was detected at a frequency of 24.8% in the AIH group; this value was higher than that in the other groups. With regard to IgG quantification, the proportion of IgG-high group was high in the AIH group than in the other groups.

**Table 1:** Patient demographic features according to diagnosis.

		Diagnosis			
		Autoimmune hepatitis (n = 117)	Nonalcoholic steatohepatitis (n = 192)	Viral hepatitis (n = 320)	Other hepatitis (n = 742)
Sex (N, %)	Male	29 (24.8%)	87 (45.3%)	117 (36.6%)	269 (36.3%)
	Female	88 (75.2%)	105 (54.7%)	203 (63.4%)	473 (63.7%)
Age (mean±SD)		56.0±14.2	49.7±15.4	53.9±14.5	54.8±16.3
ANA	Negative	17 (19.8%)	136 (76%)	193 (67%)	429 (63.6%)
	Positive	69 (80.2%)	43 (24%)	95 (33%)	246 (36.4%)
Anti-SMA	Negative	68 (73.9%)	155 (86.1%)	261 (88.5%)	630 (90.8%)
	Positive	24 (26.1%)	25 (13.9%)	34 (11.5%)	64 (9.2%)
p-ANCA	Negative	45 (66.2%)	139 (90.3%)	157 (84.9%)	549 (86.6%)
	Positive	23 (33.8%)	15 (9.7%)	28 (15.1%)	85 (13.4%)
Anti-SLA/LP	Negative	111 (94.9%)	188 (97.9%)	309 (96.6%)	707 (95.3%)
	Borderline	5 (4.3%)	4 (2.1%)	5 (1.6%)	30 (4%)
	Positive	1 (0.9%)	0 (0%)	6 (1.9%)	5 (0.7%)
Anti-LC-1	Negative	104 (88.9%)	185 (96.4%)	294 (91.9%)	700 (94.3%)
	Borderline	7 (6%)	3 (1.6%)	17 (5.3%)	27 (3.6%)
	Positive	6 (5.1%)	4 (2.1%)	9 (2.8%)	15 (2%)
Anti-LKM-1	Negative	117 (100%)	191 (99.5%)	315 (98.4%)	734 (98.9%)
	Borderline	0 (0%)	0 (0%)	5 (1.6%)	5 (0.7%)
	Positive	0 (0%)	1 (0.5%)	0 (0%)	3 (0.4%)
AMA-M2	Negative	84 (71.8%)	178 (92.7%)	278 (86.9%)	658 (88.7%)
	Borderline	8 (6.8%)	6 (3.1%)	8 (2.5%)	20 (2.7%)
	Positive	25 (21.4%)	8 (4.2%)	34 (10.6%)	64 (8.6%)
Anti-Ro-52	Negative	85 (72.6%)	176 (91.7%)	273 (85.3%)	660 (88.9%)
	Borderline	3 (2.6%)	3 (1.6%)	11 (3.4%)	16 (2.2%)
	Positive	29 (24.8%)	13 (6.8%)	36 (11.3%)	66 (8.9%)
Anti-gp210	Negative	102 (87.2%)	185 (96.4%)	294 (91.9%)	677 (91.2%)
	Borderline	3 (2.6%)	6 (3.1%)	13 (4.1%)	24 (3.2%)
	Positive	12 (10.3%)	1 (0.5%)	13 (4.1%)	41 (5.5%)
Anti-PML	Negative	115 (98.3%)	191 (99.5%)	316 (98.8%)	739 (99.6%)
	Borderline	0 (0%)	0 (0%)	2 (0.6%)	0 (0%)
	Positive	2 (1.7%)	1 (0.5%)	2 (0.6%)	3 (0.4%)
Anti-Sp100	Negative	104 (88.9%)	191 (99.5%)	308 (96.3%)	729 (98.2%)
	Borderline	4 (3.4%)	0 (0%)	0 (0%)	4 (0.5%)
	Positive	9 (7.7%)	1 (0.5%)	12 (3.8%)	9 (1.2%)
IgG quantification	Low	30 (26.5%)	168 (89.4%)	228 (73.8%)	557 (76.5%)
	Medium	14 (12.4%)	7 (3.7%)	29 (9.4%)	71 (9.8%)
	High	69 (61.1%)	13 (6.9%)	52 (16.8%)	100 (13.7%)

### Autoantibody levels according to IgG quantification

For patients who underwent testing for IgG quantification, autoantibody levels were analyzed for three groups (IgG-low, -medium, and -high). The relationship between IgG quantification and autoantibodies was found to be significant (Table 2). When examining using Hep-2 cells, the positivity rate of ANA in the IgG-high group was 66.8%. When examining using IIF, the positivity rate of p-ANCA was 10% in the IgG-low group and 32% in the IgG-high group (approximately three times higher). The AMA-M2 positivity rate was 7% in the IgG-low group and 21% in the IgG-high group (approximately three times higher). The Ro-52 positivity rate was 6% in the IgG-low group and 27% in the IgG-high group (more than four times higher).

**Table 2:** Prevalence of autoantibodies according to IgG quantification.

		IgG Quantification			p-value
		Low	Medium	High	
ANA	Negative	642	61	66	<0.00001
	Positive	267	49	133	
Anti-SMA	Negative	829	97	182	0.426143
	Positive	102	16	28	
p-ANCA	Negative	688	78	120	<0.00001
	Positive	79	15	57	
Anti-SLA/LP	Negative	953	113	217	0.005051
	Borderline	26	6	11	
	Positive	4	2	6	
Anti-LC-1	Negative	937	108	208	0.001515
	Borderline	27	8	16	
	Positive	19	5	10	
Anti-LKM-1	Negative	972	121	231	N/A
	Borderline	7	0	3	
	Positive	4	0	0	
AMA-M2	Negative	892	104	174	<0.00001
	Borderline	26	3	12	
	Positive	65	14	48	
Anti-Ro-52	Negative	900	102	164	<0.00001
	Borderline	20	7	6	
	Positive	63	12	64	
Anti-gp210	Negative	912	114	202	0.015751
	Borderline	28	4	12	
	Positive	43	3	20	
Anti-PML	Negative	978	120	230	0.277044
	Borderline	1	0	1	Except PML borderline
	Positive	4	1	3	
Anti-Sp100	Negative	963	119	217	0.000032
	Borderline	6	1	1	
	Positive	14	1	16	

### Groups meeting the three set conditions

As mentioned in the Methods, we set three conditions to determine the AIH candidate group among patients with no liver biopsies: no viral hepatitis, high IgG levels, and positivity for at least one autoantibody. Patients were classified into groups by scoring each condition as one point, after which the diagnosis and ratio of each antibody were analyzed (Table 3). The scoring system matched with the patient’s diagnosis; of the patients who received three points, 56 (38.6%) were diagnosed as having AIH, and of the ones who received two points, 51 (10.5%) were diagnosed as having AIH. Patients who scored three points had higher positivity rates for most autoantibodies than those of patients in the other groups.

**Table 3:** Results of variables examined by modified simplified score.

	Score				
		3	2	1	0
Sex	Male	34 (23.4%)	159 (32.7%)	245 (41.2%)	64 (43.8%)
	Female	111 (76.6%)	327 (67.3%)	349 (58.8%)	82 (56.2%)
Age		58.6 (14.8)	56.7 (15.1)	51.4 (15.9)	50.6 (15.1)
Diagnosis	Autoimmune hepatitis	56 (38.6%)	51 (10.5%)	10 (1.7%)	0 (0.0%)
	Nonalcoholic steatohepatitis	9 (6.2%)	74 (15.2%)	109 (18.4%)	0 (0.0%)
	Viral hepatitis	0 (0.0%)	43 (8.8%)	132 (22.2%)	145 (99.3%)
	Other hepatitis	80 (55.5%)	318 (65.4%)	343 (57.7%)	1 (0.7%)
ANA	Negative	22 (17.5%)	175 (38.7%)	451 (86.2%)	127 (100.0%)
	Positive	104 (82.5%)	277 (61.3%)	72 (13.8%)	0 (0.0%)
Anti-SMA	Negative	114 (85.7%)	351 (77.7%)	517 (95.0%)	132 (100.0%)
	Positive	19 (14.3%)	101 (22.3%)	27 (5.0%)	0 (0.0%)
p-ANCA	Negative	72 (61.5%)	307 (77.7%)	431 (96.0%)	80 (100.0%)
	Positive	45 (38.5%)	88 (22.3%)	18 (4.0%)	0 (0.0%)
Anti-SLA/LP	Negative	137 (94.5%)	462 (95.1%)	570 (96.0%)	146 (100.0%)
	Borderline	7 (4.8%)	14 (2.9%)	23 (3.9%)	0 (0.0%)
	Positive	1 (0.7%)	10 (2.1%)	1 (0.2%)	0 (0.0%)
Anti-LC-1	Negative	129 (89.0%)	447 (92.0%)	569 (95.8%)	138 (94.5%)
	Borderline	9 (6.2%)	18 (3.7%)	19 (3.2%)	8 (5.5%)
	Positive	7 (4.8%)	21 (4.3%)	6 (1.0%)	0 (0.0%)
Anti-LKM-1	Negative	143 (98.6%)	481 (99.0%)	588 (99.0%)	145 (99.3%)
	Borderline	2 (1.4%)	1 (0.2%)	6 (1.0%)	1 (0.7%)
	Positive	0 (0.0%)	4 (0.8%)	0 (0.0%)	0 (0.0%)
AMA-M2	Negative	101 (69.7%)	398 (81.9%)	555 (93.4%)	144 (98.6%)
	Borderline	6 (4.1%)	20 (4.1%)	14 (2.4%)	2 (1.4%)
	Positive	38 (26.2%)	68 (14.0%)	25 (4.2%)	0 (0.0%)
Anti-Ro-52	Negative	94 (64.8%)	402 (82.7%)	555 (93.4%)	143 (97.9%)
	Borderline	0 (0.0%)	15(3.1%)	15 (2.5%)	3 (2.1%)
	Positive	51 (35.2%)	69 (14.2%)	24 (4.0%)	0 (0.0%)
Anti-gp210	Negative	119 (82.1%)	434 (89.3%)	562 (94.6%)	143 (97.9%)
	Borderline	7 (4.8%)	17 (3.5%)	19 (3.2%)	3 (2.1%)
	Positive	19 (13.1%)	35 (7.2%)	13 (2.2%)	0 (0.0%)
Anti-PML	Negative	143 (98.6%)	480 (98.8%)	592 (99.7%)	146 (100.0%)
	Borderline	0 (0.0%)	1 (0.2%)	1 (0.2%)	0 (0.0%)
	Positive	2 (1.4%)	5 (1.0%)	1 (0.2%)	0 (0.0%)
Anti-Sp100	Negative	133 (91.7%)	467 (96.1%)	586 (98.7%)	146 (100.0%)
	Borderline	1 (0.7%)	6 (1.2%)	1 (0.2%)	0 (0.0%)
	Positive	11 (7.6%)	13 (2.7%)	7 (1.2%)	0 (0.0%)
IgG quantification	High	145 (100.0%)	79(16.5%)	10 (1.7%)	0 (0.0%)
	Medium	0 (0.0%)	55(11.5%)	49 (8.5%)	17 (12.2%)
	Low	0 (0.0%)	344(72.0%)	517 (89.8%)	122 (87.8%)

### Comparison of patients diagnosed according to the EASL criteria

We classified patients into the "Consider AIH" and "Not Considered" groups according to the EASL criteria and further classified AMA-M2–positive patients into the "AMA" group to compare AST, ALT, gamma-GT, and IgG quantification values (Table 4). We observed no significant differences in the AST, ALT, and gamma-GT values among the three groups. The IgG quantification values were three to four times higher in the "Consider AIH" and "AMA" groups than in the "Not Considered" group. The "Consider AIH" and "AMA" groups had similar IgG-low, -medium, and -high distributions.

**Table 4:** Results of liver enzyme and IgG quantification classified by AIH according to the EASL guidelines.

		Considered AIH	AMA positive	Not considered
AST	Mean	198.6 (615.6)	168.6 (232.1)	196.8 (526.3)
	Median	56	52	53
ALT	Mean	212.7 (726.8)	291.5 (516.1)	273.1 (810.9)
	Median	44	59	60
Gamma-GT	Mean	172.7 (233.7)	136.7 (197.1)	189.0 (288.0)
	Median	94	44	87
IgG quantification	Low	432 (63.2%)	21 (67.7%)	530 (85.1%)
	Medium	68 (9.9%)	3 (9.7%)	50 (8.0%)
	High	184 (26.9%)	7 (22.6%)	43 (6.9%)

## Discussion

This study aimed to determine the prevalence and positivity pattern of various antibodies and IgG using LIA and the prevalence of anti-SMA, anti-AMA, p-ANCA, and ANA using IFA. Demographic features and autoantibody status were examined according to the diagnosis. We assessed the autoantibody status according to IgG quantification and in a specific group that satisfied all three criteria (non-viral hepatitis, high IgG levels, and positivity for at least one autoantibody). Finally, patients were classified into three groups by referring to guidelines regarding EASL and AMA status, and ASL, ALT, and gamma-GT levels and IgG quantification values were compared between the three groups.

The participant pool in this study was limited to patients who had hepatitis suspected to be AIH. The classification according to the EASL criteria showed that 695 out of the 1371 patients (>50%) were suspected of having AIH [16]. This proves the validity of the recruitment condition for AIH-suspected hepatitis patients who were screened via LIA before liver biopsies were performed. Known AIH-related antibodies were mostly anti-LKM-1, anti-LC1, and anti-SLA/LP [21]. In this study, the positivity rate for the above three autoantibodies was very low; however, it was possible to successfully screen for AIH by testing for other autoantibodies. In this regard, anti-LKM-1 and anti-LC-1 are known to be related to type 2 AIH. Type 2 AIH is known to be common in children; however, in this study, all participants were adults. Thus, the study was limited in its usefulness for diagnosing type 2 AIH [18].

For the diagnosis of AIH, the original 1999 AIH score is widely used [22]. However, the introduction of simplified criteria for the diagnosis of AIH in 2008 made the diagnostic process more straightforward [20]. While these criteria place emphasis on autoimmunity, they require regional validation. For example, in the year following its introduction, validation studies were conducted in various countries, including Italy [23]. A study in Korea reported on the diagnostic accuracy of the simplified criteria in patients across a range of diagnoses, and our discussion is based on the evidence that these criteria can be effectively used to diagnose AIH, including overlapping syndromes [24].

Given the situation regarding medical care in Korea, liver biopsy could not be performed for all patients with suspected AIH. Of the 117 patients diagnosed with AIH per the clinician’s judgment, 78 were diagnosed via liver tissue biopsy, which was performed at this hospital for 76 of the 78 patients. Among these 76 patients, 57 (75%) showed histological findings typical of AIH or compatible with AIH. Among 19 patients without pathologically suspected AIH, 14 were suspected to have AIH according to our analyses. Additionally, among the patients with pathological compatibility for AIH, 43 were suspected to have AIH. Among the patients with the typical pathology of AIH, seven were suspected to have AIH.

Anti-SLA/LP is known to be associated with type 1 AIH but is only detected in 10–30% of AIH patients, although its specificity is 99%. According to the EASL and AASLD guidelines, several antibodies have been recognized to be associated with AIH [16, 18]. We classified patients by referring to a case-based algorithm in EASL for patients suspected of having AIH or drug-induced liver injury. We further classified positive factors in AMA antibodies, including in AASLD as AMA groups, although these are not included in EASL. Considering a 3–4-fold higher IgG quantification level in the "Consider AIH" and the "Not Considered" groups, it is appropriate to screen for autoantibodies first. Additionally, the EASL guidelines also require IgG to be tested for patient inclusion in the “Consider AIH” group. We concur that IgG levels will play an important role as a confirmatory parameter, given that the IgG-high group constituted less than 30% of the “Consider AIH” group.

We observed AMA-M2 positivity in 21.4% of patients diagnosed with AIH. In addition, AMA-M2 was positive in 20.5% of patients in the IgG-high group and in 26.0% of patients satisfying our three conditions. In addition, via classification according to EASL algorithm, it was found that the IgG quantification distribution was similar in the "Considered AIH" and "AMA positive" groups. In a Japanese study, AMA-M2 was investigated in ten patients, among whom six (60%) showed AMA-M2 positivity; however, the titer was lower than that observed in PBC and was entirely decreased in three followed-up patients [8]. In other Japanese studies, 14 out of 41 patients (31%) were AMA-positive, and clinical features and histological findings of AIH were not affected by the AMA status [25]. Meanwhile, our results were consistent with those of a Canadian study that reported that 15 (11.9%) out of 126 people diagnosed with AIH were positive for AMA [7]. In the above study, all patients had symptoms of AIH; however, progression to PBC did not occur. In contrast, when AMA-positive AIH patients underwent long-term follow-ups, there was a significant overlap with PBC [26]. In our study, 7 of 25 AMA-positive AIH patients were diagnosed with PBC-AIH syndrome. Thus, further studies are needed to clarify this association.

Of the patients diagnosed with AIH, 24.8% were Ro-52-positive. In addition, 27.4% of patients in the IgG-high group 34.9% of those who met the three conditions were Ro-52-positive. The Ro-52 antibody is associated with Sjögren’s syndrome; however, recent studies have shown that it is also associated with connective tissue disease, interstitial lung disease, AIH, and PBC [27]. A Mayo Clinic study found that 65 (38%) of 170 AIH patients had Ro-52 antibodies, with 26 having both Ro-52 and anti-SLA antibodies [28]. These findings were similar to the positivity rate in our study. Based on these similar findings, some researchers argue that further research is required to determine the presence of an association between AIH and anti-Ro autoantibodies [29]. Reportedly, anti-Ro-52-positive AIH patients have a high likelihood of developing cirrhosis and hepatic death or require liver transplantation [28]. Thus, the long-term follow-up of 29 anti-Ro-52–positive AIH patients in this study seems necessary.

In this study, 61.1% of patients diagnosed with AIH had IgG levels that exceeded 1.1 times the normal value. IgG level is an important diagnostic criterion for AIH in EASL and AASLD. IgG4-associated AIH is related to elevated IgG levels [30]. In contrast, in our study, patients were diagnosed with AIH even in the absence of IgG elevation. In AIH patients without elevated IgG levels, a delay in diagnosis is associated with poor prognosis [31]; however, when correctly diagnosed, they have high chances of undergoing successful drug treatment [32]. In addition, IgG levels are high in patients with decompensated cirrhosis and chronic liver disorder [33]. In this study, there were also patients who belonged to the IgG-high group who were not diagnosed with AIH (48.5%) and patients who were diagnosed with AIH but did not have elevated IgG levels (26.5%). Therefore, IgG levels alone cannot be used to diagnose AIH, and autoantibodies must be tested to distinguish AIH from chronic liver disorder with decompensated cirrhosis.

In this study, a total of 12 patients were positive for anti-SLA/LP autoantibodies, and one of them was considered to have AIH during the test. However, five patients were treated for rheumatoid disease during long-term follow-up. Of the 12 patients, 10 showed positivity overlap with other antibodies: six ANA, two anti-SMA, two p-ANCA, six anti-Ro-52, two anti-Sp100, two anti-LC-1, and one anti-LKM-1 cases. Similar to that in the Mayo clinic study, Ro-52 positivity was observed in six out of 12 SLA/LP-positive patients in our study [28]. Given that anti-SLA/LP autoantibodies have high specificities, and that lifetime immunosuppression is required because of a high relapse probability in anti-SLA/LP-positive AIH [34], liver biopsies and long-term follow-ups are recommended for these 12 patients.

The assessment of autoantibodies plays a crucial role in the diagnosis of AIH, with liver biopsy often being emphasized and serving as a complementary tool. Previously, analysis of various autoantibodies using different methods caused inconvenience for both the examiner and clinician. However, a key advantage of the LIA method is that it allows multiple antibodies to be tested simultaneously. This efficiency has led to an increase in the number of LIA tests performed annually at the study hospital. Previous studies have primarily focused on the ability of LIA to test multiple antibodies simultaneously, showing a passive stance regarding its role in the diagnosis of AIH. However, this study suggests that LIA plays a supportive role in the diagnosis of AIH and can be particularly important when pathological results are inconclusive.

There are several limitations in this study. First, not all hepatitis patients were tested in this study; only patients suspected of having AIH were tested. Consequently, this article is primarily useful as a report on patients suspected to have AIH and provides data for antibody analysis specific to AIH. Second, the study’s duration was fairly short; therefore, we are currently collecting more data for future studies. Data on the clinical course of the patients and results of their liver biopsies were insufficient due to the large number of patients. Thus, a future study is being planned to report specific results. Liver biopsy is an invasive test and cannot be performed in all patients with suspected AIH. Antibody tests are commonly used as screening tests, and it would be appropriate to conduct additional diagnostic evaluations based on these results.

To conclude, we identified factors and autoantibodies associated with AIH via a retrospective study. IgG and autoantibodies levels were significantly correlated with AIH but none of them were definitive markers for diagnosis. Therefore, to diagnose AIH accurately, several markers must be analyzed together. We plan to further investigate the influence of each antibody on the patient’s prognosis in future studies.

## Supporting information

S1 File Original dataset of the study. (XLSX)

## References

[1] Floreani A, Restrepo-Jiménez P, Secchi MF, De Martin S, Leung PSC, Krawitt E, et al. Etiopathogenesis of autoimmune hepatitis. J Autoimmun. 2018;95:133–43. Epub 2018/11/06. doi: 10.1016/j.jaut.2018.10.020 . 30385083

[2] Czaja AJ, Manns MP. The validity and importance of subtypes in autoimmune hepatitis: a point of view. Am J Gastroenterol. 1995;90(8):1206–11. Epub 1995/08/01. . 7639216

[3] Muratori P, Granito A, Quarneti C, Ferri S, Menichella R, Cassani F, et al. Autoimmune hepatitis in Italy: the Bologna experience. J Hepatol. 2009;50(6):1210–8. Epub 2009/04/28. doi: 10.1016/j.jhep.2009.01.020 . 19395113

[4] Islek A, Keskin H. Seronegative autoimmune hepatitis in children: a single-center experience. Acta Gastroenterol Belg. 2021;84(2):305–10. Epub 2021/07/05. doi: 10.51821/84.2.305 . 34217180

[5] Khedr MA, Salem TA, Boghdadi GM, Elharoun AS, El-Shahaway AA, Atallah HR, et al. Seronegative autoimmune hepatitis in children: A real diagnostic challenge. Wien Klin Wochenschr. 2022;134(5–6):195–201. Epub 2021/07/21. doi: 10.1007/s00508-021-01907-x . 34283299

[6] Zauli D, Ghetti S, Grassi A, Descovich C, Cassani F, Ballardini G, et al. Anti-neutrophil cytoplasmic antibodies in type 1 and 2 autoimmune hepatitis. Hepatology. 1997;25(5):1105–7. Epub 1997/05/01. doi: 10.1002/hep.510250510 . 9141425

[7] Thein HH, Yi Q, Dore GJ, Krahn MD. Estimation of stage-specific fibrosis progression rates in chronic hepatitis C virus infection: a meta-analysis and meta-regression. Hepatology. 2008;48(2):418–31. Epub 2008/06/20. doi: 10.1002/hep.22375 . 18563841

[8] Tomizawa M, Shinozaki F, Fugo K, Motoyoshi Y, Sugiyama T, Yamamoto S, et al. Anti-mitochondrial M2 antibody-positive autoimmune hepatitis. Exp Ther Med. 2015;10(4):1419–22. Epub 2015/12/02. doi: 10.3892/etm.2015.2694 . 26622500 PMC4578094

[9] Wies I, Brunner S, Henninger J, Herkel J, Kanzler S, Meyer zum Büschenfelde KH, et al. Identification of target antigen for SLA/LP autoantibodies in autoimmune hepatitis. Lancet. 2000;355(9214):1510–5. Epub 2000/05/09. doi: 10.1016/s0140-6736(00)02166-8 . 10801173

[10] Martini E, Abuaf N, Cavalli F, Durand V, Johanet C, Homberg JC. Antibody to liver cytosol (anti-LC1) in patients with autoimmune chronic active hepatitis type 2. Hepatology. 1988;8(6):1662–6. Epub 1988/11/01. doi: 10.1002/hep.1840080632 . 3192182

[11] Cancado EL, Abrantes-Lemos CP, Terrabuio DR. The importance of autoantibody detection in autoimmune hepatitis. Front Immunol. 2015;6:222. Epub 2015/06/02. doi: 10.3389/fimmu.2015.00222 . 26029208 PMC4429613

[12] Kikuchi J, Hashizume M, Kaneko Y, Yoshimoto K, Nishina N, Takeuchi T. Peripheral blood CD4(+)CD25(+)CD127(low) regulatory T cells are significantly increased by tocilizumab treatment in patients with rheumatoid arthritis: increase in regulatory T cells correlates with clinical response. Arthritis research & therapy. 2015;17(1):10. Epub 2015/01/22. doi: 10.1186/s13075-015-0526-4 . 25604867 PMC4332922

[13] Huang C, Han W, Wang C, Liu Y, Chen Y, Duan Z. Early Prognostic Utility of Gp210 Antibody-Positive Rate in Primary Biliary Cholangitis: A Meta-Analysis. Dis Markers. 2019;2019:9121207. Epub 2019/11/19. doi: 10.1155/2019/9121207 . 31737133 PMC6815635

[14] Papamichalis PA, Zachou K, Papamichali RA, Ioannou M, Gatselis NK, Dalekos GN, et al. Promyelocytic Leukemia Antigen Expression: a Histological Marker for Primary Biliary Cholangitis Diagnosis? J Transl Int Med. 2021;9(1):43–51. Epub 2021/04/15. doi: 10.2478/jtim-2021-0008 . 33850801 PMC8016348

[15] Szostecki C, Guldner HH, Netter HJ, Will H. Isolation and characterization of cDNA encoding a human nuclear antigen predominantly recognized by autoantibodies from patients with primary biliary cirrhosis. J Immunol. 1990;145(12):4338–47. Epub 1990/12/15. . 2258622

[16] EASL Clinical Practice Guidelines: Autoimmune hepatitis. J Hepatol. 2015;63(4):971–1004. Epub 2015/09/06. doi: 10.1016/j.jhep.2015.06.030 . 26341719

[17] Galaski J, Weiler-Normann C, Schakat M, Zachou K, Muratori P, Lampalzer S, et al. Update of the simplified criteria for autoimmune hepatitis: Evaluation of the methodology for immunoserological testing. J Hepatol. 2021;74(2):312–20. Epub 2020/07/31. doi: 10.1016/j.jhep.2020.07.032 . 32730794

[18] Mack CL, Adams D, Assis DN, Kerkar N, Manns MP, Mayo MJ, et al. Diagnosis and Management of Autoimmune Hepatitis in Adults and Children: 2019 Practice Guidance and Guidelines From the American Association for the Study of Liver Diseases. Hepatology. 2020;72(2):671–722. Epub 2019/12/22. doi: 10.1002/hep.31065 . 31863477

[19] Saito H, Takahashi A, Abe K, Okai K, Katsushima F, Monoe K, et al. Autoantibodies by line immunoassay in patients with primary biliary cirrhosis. Fukushima J Med Sci. 2012;58(2):107–16. Epub 2012/12/15. doi: 10.5387/fms.58.107 . 23237866

[20] Hennes EM, Zeniya M, Czaja AJ, Parés A, Dalekos GN, Krawitt EL, et al. Simplified criteria for the diagnosis of autoimmune hepatitis. Hepatology. 2008;48(1):169–76. Epub 2008/06/10. doi: 10.1002/hep.22322 . 18537184

[21] Zhao Y, Yan HP, Tan YF, Feng X, Liu Y, Cui D, et al. [The significance of anti-soluble liver antigen/liver-pancreas in diagnosing and typing autoimmune hepatitis]. Zhonghua Gan Zang Bing Za Zhi. 2007;15(4):283–6. Epub 2007/04/26. . 17456317

[22] Alvarez F, Berg PA, Bianchi FB, Bianchi L, Burroughs AK, Cancado EL, et al. International Autoimmune Hepatitis Group Report: review of criteria for diagnosis of autoimmune hepatitis. J Hepatol. 1999;31(5):929–38. Epub 1999/12/02. doi: 10.1016/s0168-8278(99)80297-9 . 10580593

[23] Muratori P, Granito A, Pappas G, Muratori L. Validation of simplified diagnostic criteria for autoimmune hepatitis in Italian patients. Hepatology. 2009;49(5):1782–3; author reply 3. Epub 2009/04/30. doi: 10.1002/hep.22825 . 19402135

[24] Lee Y, Kim Y, Kim S, Lim J, Won J, Jang J, et al. Diagnostic Value and Utility of the Simplified International Autoimmune Hepatitis Group (IAIHG) Criteria for Autoimmune Hepatitis in Korea. Korean J Med. 2011;81(3):340–50.

[25] Nezu S, Tanaka A, Yasui H, Imamura M, Nakajima H, Ishida H, et al. Presence of antimitochondrial autoantibodies in patients with autoimmune hepatitis. J Gastroenterol Hepatol. 2006;21(9):1448–54. Epub 2006/08/17. doi: 10.1111/j.1440-1746.2006.04434.x . 16911691

[26] Dinani AM, Fischer SE, Mosko J, Guindi M, Hirschfield GM. Patients with autoimmune hepatitis who have antimitochondrial antibodies need long-term follow-up to detect late development of primary biliary cirrhosis. Clin Gastroenterol Hepatol. 2012;10(6):682–4. Epub 2012/03/01. doi: 10.1016/j.cgh.2012.02.010 . 22366178

[27] Ferreira JP, Almeida I, Marinho A, Cerveira C, Vasconcelos C. Anti-ro52 antibodies and interstitial lung disease in connective tissue diseases excluding scleroderma. ISRN Rheumatol. 2012;2012:415272. Epub 2012/05/09. doi: 10.5402/2012/415272 . 22567412 PMC3328145

[28] Montano-Loza AJ, Shums Z, Norman GL, Czaja AJ. Prognostic implications of antibodies to Ro/SSA and soluble liver antigen in type 1 autoimmune hepatitis. Liver Int. 2012;32(1):85–92. Epub 2011/07/13. doi: 10.1111/j.1478-3231.2011.02502.x . 21745277

[29] Devesa IJF, Ferrando Ginestar J, Bustamante Balén M, Ortuño Cortés J, Borghol Hariri A, Ramos Níguez JA, et al. [Autoimmune hepatitis and anti-Ro antibodies. Is there a link?]. Gastroenterol Hepatol. 2003;26(8):475–9. Epub 2003/10/10. . 14534019

[30] Yada N, Kudo M, Chung H, Watanabe T. Autoimmune hepatitis and immunoglobulin G4-associated autoimmune hepatitis. Dig Dis. 2013;31(5–6):415–20. Epub 2013/11/28. doi: 10.1159/000355238 . 24281014

[31] Gatselis NK, Zachou K, Koukoulis GK, Dalekos GN. Autoimmune hepatitis, one disease with many faces: etiopathogenetic, clinico-laboratory and histological characteristics. World J Gastroenterol. 2015;21(1):60–83. Epub 2015/01/13. doi: 10.3748/wjg.v21.i1.60 . 25574080 PMC4284362

[32] Hartl J, Miquel R, Zachou K, Wong GW, Asghar A, Pape S, et al. Features and outcome of AIH patients without elevation of IgG. JHEP Rep. 2020;2(3):100094. Epub 2020/04/14. doi: 10.1016/j.jhepr.2020.100094 . 32280942 PMC7139106

[33] Fallatah HI, Akbar HO. Elevated serum immunoglobulin G levels in patients with chronic liver disease in comparison to patients with autoimmune hepatitis. Libyan J Med. 2010;5. Epub 2010/01/01. doi: 10.3402/ljm.v5i0.4857 . 21483590 PMC3071169

[34] Zachou K, Weiler-Normann C, Muratori L, Muratori P, Lohse AW, Dalekos GN. Permanent immunosuppression in SLA/LP-positive autoimmune hepatitis is required although overall response and survival are similar. Liver Int. 2020;40(2):368–76. Epub 2019/10/19. doi: 10.1111/liv.14280 . 31626725

